# ATMP development and pre-GMP environment in academia: a safety net for early cell and gene therapy development and manufacturing

**DOI:** 10.1016/j.iotech.2022.100099

**Published:** 2022-10-06

**Authors:** D.N. Silva, M. Chrobok, G. Ahlén, P. Blomberg, M. Sällberg, A. Pasetto

**Affiliations:** 1 Department of Laboratory Medicine, Karolinska Institutet, Stockholm, Sweden; 2 Vecura, Karolinska Cell Therapy Center, Karolinska University Hospital, Stockholm, Sweden; 3 Section for Cell Therapy, Radiumhospitalet, Oslo University Hospital, Oslo, Norway

**Keywords:** pre-GMP, ATMP, process development, regulatory support

## Abstract

Advanced therapy medicinal products (ATMP) are medicines for human use that are based on genes, cells or tissues. Over the past years, an increasing number of ATMP entered the market for treatment of cancer, genetic disorders, skeletal defects and metabolic diseases. However, the ATMP production methods often change from the initial concept to commercialization. This change is needed to improve the manufacturing feasibility for scaling up or scaling out. Moreover, the production must adhere to current good manufacturing practices (GMP), and needs to follow a risk-based approach, which often is challenging to implement due to the novelty of the products. Since most of the early ATMP development is done in academia, an environment that is not familiar with regulatory requirements for ATMP production in GMP, the initial manufacturing choice for pre-clinical studies is usually very different from what is required for clinical use. This leads to a lengthy production process optimization, unnecessary repetition of experiments and ultimately waste of funding. This consideration prompted us to provide an intermediate step between early ATMP production in research settings to GMP manufacturing. We built a dedicated facility, and we called this environment ‘pre-GMP’ to highlight that it is a step toward preparation to GMP manufacturing. This environment supports process development and provides a manufacturing fitness room before transferring to GMP suites. This paper addresses the relevance of pre-GMP, underlining the advantages and the possible disadvantages of this additional framework that may be key in accelerating the pace of ATMP toward clinic.

## Highlights

• Challenges faced during ATMP development in academia.
• Experience in providing support to ATMP development by building and operating a pre-GMP core facility.
• Consideration of the benefits of the pre-GMP model for other academic institutions.

## ATMP and their challenges

Advanced therapy medicinal products (ATMP) are medicines for human use that are based on genes, tissues or cells. They are a group of innovative medicines, both in their manufacturing processes and in the mechanism of action, which offer new opportunities in the treatment of different diseases.^1^

ATMP can be classified into three major groups: (i) gene therapy medicines in which genetic sequences are designed to modify, control, inhibit or overexpress a specific target gene sequence^2^; (ii) somatic cell therapy medicines which can be highly variable, in which the cells can be from autologous or allogeneic origin and can be in different stages of differentiation such as adult cells, fully differentiated, self-renewing stem cells or progenitor cells^3^; (iii) tissue-engineered medicines which are products that have in their content cells or tissues that have been modified with a combination of different techniques of cellular and molecular biology, biomaterials and engineering, with their goal being to repair, regenerate or replace human tissue to restore tissue function or replace unhealthy tissues. In addition, some ATMP may contain one or more medical devices as an integral part of the medicine, which are referred to as combined ATMP (Table 1).

**Table 1:** Marketed ATMP authorized by EMA and FDA

ATMP classification	Product	Manufacturer	Agency
Somatic cell therapy	Allogeneic cultured keratinocytes and fibroblasts in bovine collagen	Organogenesis	FDA
	Allogeneic T cells genetically modified with a retroviral vector encoding for a truncated form of the human low-affinity nerve growth factor receptor (DLNGFR) and the herpes simplex I virus thymidine kinase (HSV-TK Mut2)	MolMed Spa	EMA
	Darvadstrocel	Takeda Pharma	EMA
	Hematopoietic progenitor cell cord blood	Cleveland Cord Blood Center	FDA
		SSM Cardinal Glennon Children’s Medical Center	
		Bloodworks	
		Clinimmune Labs, University of Colorado Cord Blood Bank	
		Duke University School of Medicine	
		LifeSouth Community Blood Centers	
		New York Blood Center	
		MD Anderson Cord Blood Bank	
	Sipuleucel-T	Dendreon	FDA
	Alofisel	Tigenix	EMA
	Holoclar	Chiesi farmaceutici	EMA
Gene therapy	Autologous CD34+-enriched cell fraction that contains CD34+ cells transduced with retroviral vector that encodes for the human adenosine deaminase (ADA) cDNA sequence from human hematopoietic stem/progenitor (CD34+) cells	GlaxoSmithKline	EMA
	Imlygic	Amgen	EMA
	Axicabtagene ciloleucel	Gilead Sciences	EMA
			FDA
	Talimogene laherparepvec	Amgen	EMA
			FDA
	Tisagenlecleucel	Novartis	EMA
			FDA
	Luxturna	Spark Therapeutics	EMA
			FDA
	Strimvelis	Orchard therapeutics	EMA
	Zynteglo	Bluebird bio	EMA
Tissue-engineered products	Autologous cultured chondrocytes on a porcine collagen membrane-specific marker proteins	Chiesi Farmaceutici	EMA
	Spheroids of human autologous matrix-associated chondrocytes	Co.Don AG	EMA

ATMP, advanced therapy medicinal products; EMA, European Medicines Agency; FDA, Food and Drug Administration.

Scientific knowledge on gene and cell-based therapy products is rapidly expanding. Over the years, an increasing number of ATMP clinical trials are conducted for treatment of cancer, genetic disorders, cartilage defects and metabolic diseases as they have potential curative outcomes and also a long-lasting therapeutic effect.^4^

To ensure that reliable data are generated on these complex products, about 1788 ATMP have been examined in clinical studies on different applications. A recent search on ClinicalTrials.gov revealed that 1093 clinical studies are exploring immune-based therapies with 147 in adoptive cell therapy using T-cell receptors (TCR)-engineered T cells,^5^ 526 studies with chimeric antigen receptor T-cell therapy,^6^ 120 investigating the potential of cytotoxic T lymphocytes^7^ and about 300 studies using natural killer cells.^8^ For somatic cell-based therapy, ∼700 studies are being developed, including 683 studies with mesenchymal stromal cells^9^ and 15 studies using fibroblasts.^10^

Despite this significant number of projects, the number of ATMP on the market is still considerably low. Oftentimes, developers face a complex regulatory and developmental landscape to bring ATMP to market and some products were withdrawn within a couple of years after their market launch. As a consequence, only few ATMP have received a European Union market authorization, and this rate of new product authorization is considered low compared to the authorization rates of other types of medicinal products.^11^

At the transition from research to the developmental stage, it is important to understand the reasons of previous ATMP failures. When facing this poor success rate, some reasons are associated with the individual product and its particular characteristics; however, there are other issues that can be attributed to the whole ATMP class. In general, most of them are related to high cost of development, quality control, regulatory requirements, starting material and donor-related aspects, qualified personnel training, infrastructure and logistic limitation.^11^

Often, ATMP are under development in academia or by micro- or small- and medium-sized enterprises which are still acquiring experience in production process development and regulatory guidelines. Because ATMP is such a complex group of products, companies are facing challenges from manufacturing and product quality to the non-clinical phases up to the clinical program and data report. This leads to a high number of hurdles including rejections during the stages of approval by regulatory agencies and evaluation for marketing authorization.

The high cost of ATMP development and manufacturing limits affordability for public and private payers which will reduce patient access to treatment. An important component of the high cost is attributed to the production itself. It is not uncommon for ATMP to require custom-made good manufacturing practices (GMP)-grade reagents that are usually not possible to be mass-produced; this contributes negatively not only to the total cost of the product but also to the difficulties in standardization, qualification and supply of the materials. The manufacturing process is often very complex, characterized by multiple steps and techniques that require highly skilled operators; this can be a problem when the ATMP should be produced in different locations, both in terms of cost and also in terms of time required for training the staff. A possible solution to these problems is the introduction of automated systems, possibly fully closed, that can improve the process robustness and scalability while maintaining strict adherence to GMP and regulatory guidelines. The implementation of such automated systems can be very difficult at the early stages of ATMP development, due to the lack of flexibility of most of the automated equipment available now on the market and their high cost. However, testing this type of solutions should be encouraged, and we will discuss in the following sections how the pre-GMP can also provide support on this matter.

Currently, regulatory and policy initiatives focus on encouraging innovation and expediting review of ATMP rather than on enabling market competition and thereby ensuring affordability and availability of these new therapies.^12^^,^^13^ This means that reducing the cost while maintaining quality will become crucial as the demand for these types of products increases. We believe that the introduction of a pre-GMP step during ATMP development can help in decreasing the overall cost for production of this class of products. Among the benefits derived from the use of a pre-GMP laboratory is the possibility to test and optimize ATMP production methods in an environment that mimics GMP conditions but without the high cost of a certified clean room; another benefit derives from the access to competent staff that can provide not only technical help but also regulatory advice, supporting the investigators to achieve approval in a shorter timeframe.

The steps that involve quality control of ATMP represent another challenge for process development and approval. Tests for characterization that are needed to determine content, purity and potency are considered critical for clinical use and consequent approval by regulatory agencies. In addition, lack of biodistribution assessment, germline transmission as well as tumorigenicity studies are deficiencies frequently observed during the stages of non-clinical development.^14^ Sometimes, the manufacturing process needs to be modified to achieve the standards of quality control or to be compliant to regulatory requirements. In this sense, related safety issues or even changes in the supplies for production are recurrent and make the process development longer than expected.

Considering issues related to starting material and donor-related aspects are also relevant hurdles for ATMP development. Donor variability and differences in the starting material can substantially interfere in the characterization of the product, especially for autologous cell-based therapies. From a clinical perspective, this complicates patient selection and prediction of response. Besides that, ATMP frequently present a pleiotropic mechanism of action that is not necessarily directly linked to the nature of the product but can be influenced by patient-related factors.^15^^,^^16^

### Infrastructure issues (facilities, manufacturing, staff, logistic)

A contentious issue is arising regarding infrastructure and trained personnel for ATMP manufacturing. Training for achievement of technical and GMP expertise of all the personnel responsible for the execution of the construction work and qualification activities in the manufacture of ATMP is essential but currently lacking. This shortage of competent qualified persons also leads to a very significant added cost in ATMP manufacture. In fact, these products usually demand experience in multiple techniques to manage cell culture, sterile techniques, quality controls and release criteria.^17^^,^^18^

In addition, infrastructure is considered an important factor for ATMP manufacturing. A GMP facility is a rigorous environment associated with a significant level of documentation at every step of the process. Furthermore, all processes must be in accordance with GMP. The design of the facility is extremely expensive because it should meet the products’ manufacturing objectives and at the same time be compliant to increasing regulatory demands and future technological advancements.^18^^,^^19^ These high costs limit the number of centers capable of developing and producing ATMP.

Regarding logistic issues, distribution of ATMP that are associated with centralized manufacturing represents a challenge for many applications. This model can increase the risk for the product integrity and cost, as distribution may also compromise the quality of the product by, for example, requiring its freezing for shipping.^20^

Accounting all these issues and considering the complexity of GMP manufacturing, we believe there is a need to reinforce the concept of pre-GMP development for ATMP, as this is a crucial step for a more solid manufacturing and clinical translation. We will discuss in the following chapters the role of pre-GMP and provide examples of its beneficial influence on ATMP development.

## PRE-GMP—WHERE AND WHEN?

The pharmaceutical industry has, since a long time, recognized the value of pre-GMP for late developmental steps and early manufacturing. This value is mostly related to the advantage of having the opportunity to de-risk the manufacturing process before approaching large-scale production. The concept of a pre-GMP facility can also be used as a testbed for innovation and training site for new staff. Most of the ATMP therapeutic concepts originate in academia, where there is great opportunity for innovation, but often the translational path is full of roadblocks that are responsible for the failure or delayed success of new ATMP.

At the ‘Karolinska Institutet’, we opened in 2019 a new facility called pre-GMP, with the scope of providing support to academic researchers and small industry in their path in translating methods and processes to established GMP manufacturing for clinical trials. We designed and operated this new facility to offer solutions to the main roadblocks that we identified in ATMP development. In the following paragraphs, we will describe the problems that we encountered and the goals that we achieved.

### Pre-GMP: how should it look like?

The first roadblock that is often encountered in early ATMP development is the availability of appropriate laboratory space. Very often the original idea for a new ATMP is conceptualized in a research group and tested in a basic research environment with research-grade reagents. This environment is ideal to develop the early proof-of-concept, but it cannot provide the adequate quality system and controlled space that is required for manufacturing of products dedicated to human use.

**Figure 1:**
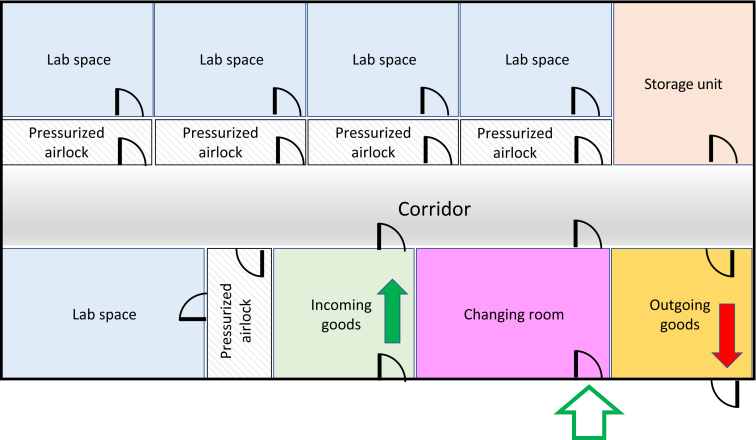
**Pre-GMP facility map.** Example of a pre-GMP facility that can accommodate multiple projects and has dedicated space for receiving goods (incoming goods), storage, changing clothes and waste disposal (outgoing goods). GMP, good manufacturing practice.

The pre-GMP facility at the ‘Karolinska Institutet’ was designed to provide a separate space for scientists where they could further test their product in an environment as close as possible to a GMP-classified environment, but without the high cost associated with GMP. The facility has restricted access, and this ensures that only authorized personnel can enter. Inside the facility there are five different rooms, with pressurized airlocks and further restricted access. This measure allows for multiple projects to be accommodated in parallel without risking contamination or bridges in confidentiality. Additionally, the facility also has a changing room to mirror the clothing and behavior requirement of GMP, a room for receiving of goods and a room for outgoing goods (Figure 1). This controlled space can be used for multiple purposes. Beside the production process development itself, the rooms in the facility can host training sessions for staff and be the meeting ground between scientists and GMP personnel for tech transfer. It is important to highlight that the rooms in the facility are not classified according to GMP cleanroom grades (e.g. A, B, C, D) and can only be used for testing of manufacturing processes and not for the actual ATMP production.

Building new facilities is a cost for universities and we understand the difficulties related to gathering funding to support this new pre-GMP concept. We took advantage of refurbishment projects occurring at ‘Karolinska Institutet’ to build a completely new facility, and although we recommend a dedicated space for pre-GMP, other solutions such as repurposing older laboratories could also satisfy the scope at a significant lower cost.

### The importance of equipment

Having the appropriate equipment in place may seem an obvious demand to conduct scientific research, but the quality standards that are required for GMP manufacturing are often very far from the daily routines of basic laboratories. Therefore, a common roadblock for scientists is the inability to carry out their production process development with the same instrumentations that will be later used for GMP manufacturing. For example, basic aspects such cell counting can make a big difference during process development; in fact, trying to optimize and standardize a process to achieve a certain cell number when the cell number itself is not reliable will inevitably cause delays when the process needs to be transferred to a GMP environment.

Equipment utilized in GMP manufacturing could have specific design requirements to ensure compliance; for example, surfaces that can be disinfected or sterilized, material with limited particle emission, redundancy in measuring crucial parameters; moreover, calibration and maintenance must be carried out periodically, and detailed logs need to be kept. Such attention to the equipment is necessary, but it is also associated with high cost. To also support scientists on this aspect, we harmonized the equipment in the pre-GMP facility with the GMP manufacturing unit ‘Vecura’ present at the ‘Karolinska Institutet’ University Hospital. We also invested in state-of-the-art automated equipment to offer our users the opportunity to test these instruments without having to commit to purchase. In this context, one study that was conducted in our facility has been recently published,^21^ highlighting how academia can be at the front line for production process development in an efficient and cost-effective way.

The availability of funding to provide this type of equipment could be a limiting factor for many academic institutions. We believe that this gap could be filled by a positive collaborative attitude with the supplier’s industry. Pre-GMP facilities in academia could also be used to accommodate demo instruments or prototypes, promoting scientific collaborations between scientists and industry.

### Support beyond the infrastructure

Another major roadblock that can seriously delay ATMP development is the lack of regulatory support. ATMP products are often complex and have innovative aspects that are difficult to frame from a regulatory perspective. Most scientists in academia have received a traditional training that may not include knowledge of drug manufacturing regulations, and it can become overwhelming to have to deal with regulatory requirements while also pursuing the optimization of an ATMP manufacturing process.

We therefore envision the pre-GMP also as competence hub, where scientists can receive support and guidance to prepare their regulatory applications. To achieve this goal, we recruited staff for the facility that had experience with both the research environment and the GMP environment. Staff with this profile is still quite rare, so we made it our mission to train students and professionals coming to our facility so that at least some of the skills and competences could be transferred. Although this could be a temporarily solution for the projects that come to the facility, we strongly believe that basic training in GMP and regulatory concepts should become the norm in medical universities.

## Pre-GMP environment for ATMP future development

The translation of cell therapy toward the clinic is not generally dissimilar to other medicinal products and involves a series of challenges, as mentioned earlier. Since most cell-based products have emerged from academia, they may miss the correct roadmap to achieve the clinical use and market authorization.

In some cases, GMP facilities in hospitals/universities spin out from research and development laboratories, and so may carry the failure roots of early academic research. Thus, it does require a cultural step in the direction of cell manufacturing, cell culture standardization and quality control/assurance in early phase development. In this sense, having a pre-GMP concept/space in academia preferably in connection to manufacturing sites in hospitals can be a catalyst for acceleration of ATMP entering clinical trials and market.

The pre-GMP environment favors the future development of ATMP in different steps that affects clinical translation. The first step is related to concept evaluation in which the creation of ‘proof-of-concept’ can be developed in a context closer to clinical-grade manufacturing conditions. *In vitro* and *in vivo* studies can be carried out to define a cellular product’s desired characteristics, as well as cell toxicity, cell survival kinetics/distribution and toxicology studies, where cell toxicity, cell survival kinetics/distribution and toxicology studies can be carried out to define a cellular product’s desired characteristics.

The second step is a phase of production process optimization that is generally challenging for ATMP development. This is associated with the optimization of a reproducible, large-scale manufacturing process that parallels the creation of a clinical study design. This also involves translational development into a GMP laboratory to develop tools, to scale up the process, and to optimize manufacturing in which the incorporation of regulatory-grade product characterization and quality controls are required. In the case of gene therapy, for example, this is also linked with viral vector development and manufacturing. In this early phase, obstacles in the translation of products toward the clinic may be apparent, and production optimization is an important phase of development since the technologies used in early studies may not be optimized or transferrable for larger/clinical-scale cell production. Reagents such as tissue culture media and ancillary reagents utilized in the research laboratories may not be suitable for human uses, and the product features may not be robustly defined. Thus, the main goal of ATMP development in the pre-GMP environment is to identify key aspects of the product and the process that may represent a problem for subsequent clinical translation and provide support to perform the necessary changes. Taking as example gene therapy based on viral vectors, the vector comparability between research grade and GMP grade may become a relevant issue. It could be necessary to change the vector backbone and the type of promoter for safety reasons, and also the manufacturing steps need to be optimized to ensure the production of an adequate quantity of vector for use in clinical trials and they need to be compliant with regulations. Considering these aspects, a pre-GMP environment would accelerate the ATMP entering clinical trials since the infrastructure, equipment and safety aspects would be considered very early in the product development, shortening the process optimization and de-risking the subsequent large-scale manufacturing.

The third step involves methods qualification, which is considered an important aspect of ATMP clinical translation. Generally, in this step a clinically appropriate and optimized method has been defined. The pre-GMP infrastructure can be used to establish standard operating procedures, generate a qualification plan and possibly also execute pre-validation runs for dedicated products and processes.

In this phase, the planned qualification steps can be easily transferred from pre-GMP to the GMP site and generate results that can be provided to the regulatory body and to the institutional review board for approval.

**Figure 2:**
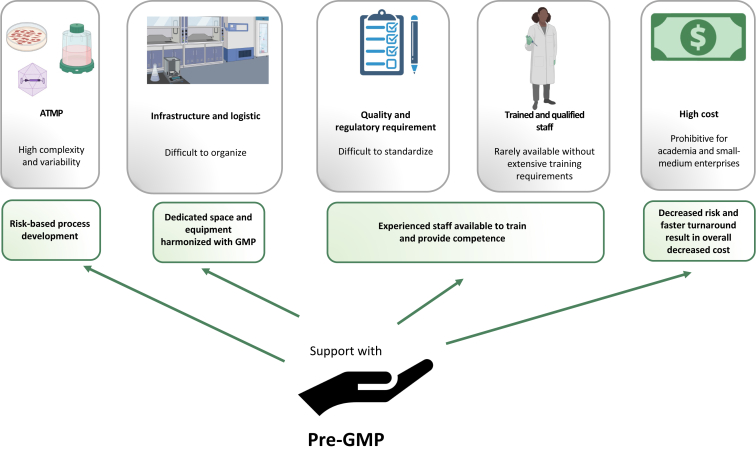
**Pre-GMP support functions.** Schematic representations of the support provided by an academic pre-GMP infrastructure and competence hub regarding the major challenges encountered by scientists developing new ATMP in an academic setting. ATMP, advanced therapy medicinal products; GMP, good manufacturing practice.

Here, we identified some of the major areas where competent and timely support could make the difference for a successful development of new ATMP, especially in academia (Figure 2). We believe that promoting the pre-GMP concept is instrumental for the advancement of the field and to achieve safe and cost-effective therapies for currently deadly diseases.
